# Heavy with child? Pregnancy status and stable isotope ratios as determined from biopsies of humpback whales

**DOI:** 10.1093/conphys/cow050

**Published:** 2016-10-18

**Authors:** Casey T. Clark, Alyson H. Fleming, John Calambokidis, Nicholas M. Kellar, Camryn D. Allen, Krista N. Catelani, Michelle Robbins, Nicole E. Beaulieu, Debbie Steel, James T. Harvey

**Affiliations:** 1 Moss Landing Marine Laboratories, Moss Landing, CA 95039, USA; 2 College of Fisheries and Ocean Sciences, University of Alaska Fairbanks, Fairbanks, AK 99775, USA; 3 Department of Paleobiology and Vertebrate Zoology, National Museum of Natural History Smithsonian Institution, Washington, DC 20013, USA; 4 Cascadia Research Collective, Olympia, WA 98501, USA; 5 Marine Mammal and Turtle Division, Southwest Fisheries Science Center, National Marine Fisheries Service, National Oceanic and Atmospheric Administration, La Jolla, CA 92037, USA; 6 Marine Mammal Institute and Department of Fisheries and Wildlife, Oregon State University, Newport, OR 97365, USA

**Keywords:** Blubber, hormones, humpback whale, pregnancy, progesterone, stable isotopes

## Abstract

Understanding reproductive rates of wild animal populations is crucially important for management and conservation. Assessing pregnancy status of free-ranging cetaceans has historically been difficult; however, recent advances in analytical techniques have allowed the diagnosis of pregnancy from small samples of blubber tissue. The primary objectives of this study were as follows: (i) to test the efficacy of blubber progesterone assays as a tool for diagnosing pregnancy in humpback whales (*Megaptera novaeangliae*); (ii) to estimate the pregnancy rate of humpback whales in Monterey Bay, California; and (iii) to investigate the relationship between stable isotopes and reproductive status of these whales. Progesterone concentrations of female whales fell into two distinct groups, allowing for diagnostic separation of pregnant and non-pregnant individuals. Pregnancy rate varied between years of the study (48.4%% in 2011 and 18.5% in 2012), but fell within the range of other estimates of reproductive success for this population. Stable carbon and nitrogen isotope ratios were examined to investigate the impacts of pregnancy on these values. Neither δ^15^N nor δ^13^C varied in a consistent way among animals of different sex or reproductive status. The relationship between δ^15^N and δ^13^C was strongly positive for male and non-pregnant female humpbacks; however, no relationship existed for pregnant whales. This difference may be indicative of the effects of pregnancy on δ^15^N, resulting from tissue synthesis and reduced excretion of nitrogenous waste, as well as on δ^13^C through increased mobilization of lipid stores to meet the energetic demands of pregnancy. Ultimately, our results support the use of blubber progesterone assays for diagnosing pregnancy in humpback whales and indicate that, when paired with other approaches (e.g. stable isotope analysis), pregnancy status can be an informative tool for addressing questions about animal physiology, ecology and population biology. This information will provide for more effective management and conservation efforts in a rapidly changing world.

## Teaser

Recent advances in analytical techniques have allowed cetacean pregnancy status to be diagnosed using reproductive hormones from biopsy samples. We tested the efficacy of blubber progesterone assays for diagnosing pregnancy in humpback whales, calculated pregnancy rates and examined the relationship between pregnancy and stable isotope ratios for these whales.

## Introduction

The ability to determine accurately fundamental biological parameters, such as birth rates, survival rates and population growth rates, is crucial to effective management of wild animal populations. However, assessing birth rates of wild cetaceans has historically been a difficult undertaking, particularly for migratory species. Additionally, birth rates alone may not provide information about the population dynamics that are crucial to population survival (Lasley and Kirkpatrick, 1991). Pregnancy rate is an important measure of fecundity and provides valuable information about animal reproduction (e.g. perinatal mortality, abortion and fetal resorption) that is not captured when only calving rate is measured (Whitten *et al*., 1992). The majority of the current knowledge regarding cetacean pregnancy has come from examination of reproductive organs recovered from dead animals (Kellar *et al*., 2006). The use of deceased animals is inherently problematical when measuring demographic parameters, however, because the factors contributing to the death of the animals (e.g. disease, predation, ship-strike, whaling) often impact certain groups within a population more heavily than others. As a result, these dead animals are unlikely to be representative of the broader populations to which they belong.

The development of remote biopsy sampling techniques has allowed for the collection of tissue samples from free-ranging cetaceans, while limiting perturbation to the animal and decreasing sampling biases that may confound the results of molecular techniques (e.g. hormone, stable isotope or genetic analyses). This method is minimally invasive, and samples can be collected with relative ease from wild cetaceans (Lambertsen, 1987). In addition, recent advances in the efficiency and efficacy of steroid hormone analyses have allowed researchers to determine cetacean pregnancy status by assessing blubber progesterone concentrations in tissue samples collected using projectile biopsy systems (Mansour *et al*., 2002; Kellar *et al*., 2006; Trego *et al*., 2013). To date, blubber progesterone assays have been used to classify pregnancy status for 10 odontocete species and two mysticete species: bowhead (*Balaena mysticetus*) and minke whales (*Balaenoptera acutorostrata*; Mansour *et al*., 2002; Trego *et al*., 2013; Kellar *et al*., 2013a). This technique has been used to generate pregnancy rate estimates for wild dolphin populations (Kellar *et al*., 2014, 2013b) and is applied in the present study, for the first time, to determine pregnancy rates in a population of mysticete, the humpback whale (*Megaptera novaeangliae*).

In addition to providing estimates of pregnancy rates, the ability to assess blubber hormone concentrations in free-ranging cetaceans allows for investigations into a wide variety of other topics related to reproduction, including measurements of baseline hormone values of different demographic groups and investigation of seasonal patterns in hormone concentrations. Such investigations may offer additional insight into cetacean life history, reproduction and mating strategies; fundamental biological understanding that remains unknown for most cetacean species. Male humpback whales exhibit seasonal changes in testosterone concentrations during breeding and non-breeding periods (Vu *et al*., 2015); however, the existence of seasonal variability in progesterone concentrations has not been studied for wild populations. Likewise, little is currently known about how progesterone concentrations change during pregnancy in wild cetaceans. This subject has been well studied for many animal species, with highly variable results (e.g. Ranilla *et al*., 1994; Sousa *et al*., 1999). Studies of captive cetaceans indicate that progesterone concentrations are relatively stable during gestation (e.g. Robeck *et al*., 2016), although it has not been confirmed how the patterns observed in captive animals relate to wild populations. With the recent advances in steroid hormone analyses, these topics and many other questions related to the physiology and ecology of cetacean reproduction can now be addressed.

The physiological impacts of pregnancy are likely to have implications that extend beyond the field of reproductive biology into other, seemingly unrelated areas of study. Stable isotope analysis, for example, is a powerful tool for studying animal diet and migratory movements (Hobson, 1999; Kelly, 2000); however, changes within the bodies of pregnant female animals are likely to alter stable isotope ratios of the tissues, potentially confounding the results of diet and movement studies (Newsome *et al*., 2010). Pregnant mammals are expected to experience a decline in their δ^15^N values as their bodies become net anabolic and excretion of nitrogenous waste is decreased (Fuller *et al*., 2004; Newsome *et al*., 2010). Likewise, δ^13^C values would be expected to decrease as pregnant females mobilize lipid stores to meet the energetic demands of pregnancy (Kelly, 2000; Kurle and Worthy, 2001). Few researchers have assessed the effects of pregnancy on stable isotope ratios for any species, and no research has been conducted on this topic for cetaceans. The results of the few studies that have addressed this subject have been mixed, with both the existence and the magnitude of these effects varying among species (Kurle, 2002; Fuller *et al*., 2004; Habran *et al*., 2010; Newsome *et al*., 2010). Thus, understanding the impacts of pregnancy on stable isotope ratios for a particular species is an important precursor to proper interpretation of the results of stable isotope-based research. Pairing this technique with investigations of reproductive status may offer unique insights into metabolism, isotopic routing and foraging during different reproductive stages and, perhaps more importantly, the combination of stable isotope analysis with studies of reproductive status may reveal confounding factors or biases related to reproduction that would otherwise remain undetected.

The primary objectives of this study were to assess the viability of blubber progesterone assays as a tool for diagnosing pregnancy in humpback whales and to estimate pregnancy rate for humpback whales in Monterey Bay, CA, USA. Additionally, we examined how progesterone concentrations varied among animals of different demographic groups and determined whether progesterone concentrations within these groups varied through time. Finally, we investigated the relationship between reproductive status and stable isotope ratios of humpback whale skin samples.

## Materials and methods

Collection of all samples and photographs was permitted under the US Marine Mammal Protection Act by the National Marine Fisheries Service (NMFS) Permit No. 15271, issued to Dr James T. Harvey, and San Jose State University Institutional Animal Care and Use Committee (IACUC) Protocol #937.

Biopsies were obtained from free-ranging whales in the coastal waters of Monterey Bay, CA, USA using a crossbow and darts fitted with 7 mm × 25 mm biopsy tips (Lambertsen, 1987). Samples were collected from 126 individual whales during two field seasons: 62 from May to November 2011 and 64 from April to July 2012. Biopsy collection was conducted opportunistically, and tissue samples were stored at −80°C pending analysis. Fluke photographs were collected for photo-identification of individual whales. Genetic sex identification (Gilson *et al*., 1998) was conducted at the Marine Mammal Institute, Oregon State University, for all sampled individuals.

**Figure 1:**
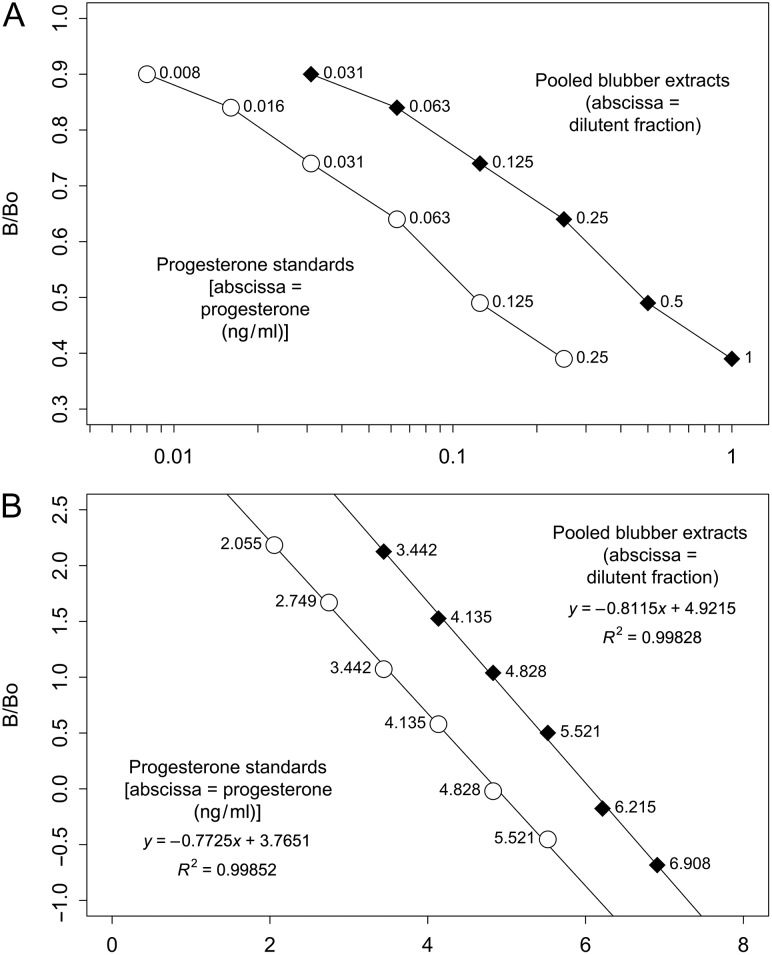
Assessment of linearity of the progesterone enzyme immunoassays (EIA) with humpback whale blubber. *B*/*B*_0_ is the ratio of optical density of bound hormone (*B*) to the maximum bound (*B*_0_). (**A**) Serial dilutions of pooled blubber extracts (filled diamonds) paralleled the slope exhibited by the EIA progesterone standards (open circles), indicating that the same antigens are being measured in the blubber and the standards, thus the assay is appropriate for use with humpback blubber tissue. (**B**) Linear regressions of logit/logarithmically transformed values of the pooled blubber extracts (filled diamonds) and the EIA progesterone standards (open circles) for testing of similarity of slopes (ANCOVA). The slopes of the linearized dilution curve of the blubber extracts were not significantly different from the assay standards (*P* = 0.121).

Blubber progesterone concentrations were determined using the extraction method as previously described by Kellar *et al*. (2015) for female whales with sufficient blubber tissue from 2011 (*n* = 31) and 2012 (*n* = 27). Blubber progesterone concentrations were also determined for a subset of males (*n* = 22) sampled in 2011, to compare progesterone concentrations between sexes. Blubber hormones were extracted using a series of biphasic chemical steps, starting with mechanical homogenization (19-040, Omni Bead Rupter-24; Omni International, Kennesaw, GA, USA) of the blubber tissue. Lipids were isolated from the tissue using a series of ethanol (100%), ethanol:acetone (4:1) and diethyl ether (100%) rinses; subsequent to each solvent rinse, the supernatant was collected. Following these solvent rinses, steroid hormones were isolated from the lipid by performing two acetonitrile and hexane rinses, in which the final acetonitrile layer was dried down and stored at −20°C prior to being run on an enzyme immunoassay (EIA). After extraction, the extracts were resuspended in 250 μl of 0.05 M phosphate-buffered saline (Sigma, St Louis, MO, USA) with 0.1% bovine serum albumin (Amresco, Solon, OH, USA) immediately prior to hormone assay. Following published methodologies used at the NMFS Southwest Fisheries Science Center (Kellar *et al*., 2015), blubber progesterone measurements were determined using a commercially available progesterone EIA kit (catalogue no. ADI-900-011; Enzo Life Sciences, Plymouth, PA, USA). A parallelism test was conducted to assess the performance of the progesterone EIA kits when used with humpback blubber extracts. This test involved serially diluting a pooled sample (*n* = 2 non-pregnant females) and analysing each dilution as well as the standard controls of the assay. In this way, it was possible to determine whether the slope of the linear decrease in the values of the serial dilutions paralleled that of the standard curve. The pooled sample extracts were analysed in triplicate starting at a 1:2 dilution and decreasing by a factor of two, ending with a 1:128 dilution. The resulting curve of the detection metric (*B*/*B*_0_) as a function of the dilution state was then compared with the standard curve using an analysis of covariance (ANCOVA), which compared slopes (Fig. 1). Blubber progesterone concentrations are presented in nanograms progesterone per gram of blubber. Pregnancy rate was defined as the percentage of females classified as pregnant (pregnant females/total females) in a given time period.

Sighting histories of female humpbacks were examined to confirm maturity status of sampled individuals. Female humpbacks typically reach maturity at 4–5 years of age (Chittleborough, 1965), thus to be conservative, only female whales previously seen with a calf or sighted at least 6 years before sampling were classified as sexually mature. Analyses of variance (ANOVAs) and Tukey's honest significant difference (HSD) *post hoc* tests were used to examine mean differences in progesterone concentrations among pregnant and mature non-pregnant females, as well as non-pregnant females of unknown maturity status, mature males and males of unknown maturity status. Linear regression analyses were used to investigate the existence of seasonal changes in progesterone concentrations among pregnant females, non-pregnant females and males in 2011. Seasonal changes were not investigated in 2012, as the duration of the sampling period was too short to capture seasonal variability in progesterone concentrations.

Preparation for stable isotope analysis was conducted at the NMFS Southwest Fisheries Science Center using the methods described by Fleming *et al*. (2015). Samples were analysed for δ^13^C and δ^15^N at the University of Florida, Gainesville Stable Isotope Geochemistry Laboratory. Results were reported per mil using delta notation, computed using the equation δ*X* = [(*R*_sample_/*R*_standard_) − 1] × 1000, where *X* represents either ^15^N or ^13^C and *R* is the ratio of ^15^N/^14^N or ^13^C/^12^C. Precision for these measurements was 0.05‰ for δ^13^C and 0.1‰ for δ^15^N. The relationship between pregnancy status and stable isotope ratios was investigated using ANOVAs and Tukey's HSD *post hoc* tests for 2011 and 2012. Additionally, we investigated the relationship between δ^13^C and δ^15^N in the tissue of individual males, non-pregnant females and pregnant females using linear regression analysis. All statistical analyses were conducted using R version 3.2.3 (R Core Team, 2016) with RStudio version 0.99.891 (RStudio Team, 2015).

## Results

The mean estimated extraction efficiency, 81.9% (range: 80.8–83.0%), was based on nine measurements of recovery of 150 ng of progesterone (P0130; Sigma-Aldrich, Saint Louis, MO, USA) spiked blubber extracts. Measured extraction efficiencies were in turn used as a correction factor applied to all blubber progesterone measurements within this study. According to the manual provided with the kit, the sensitivity of the progesterone EIA was 8.57 pg/ml.

Statistical analysis of parallelism indicated that the progesterone EIA is a viable method of detecting hormone concentration within humpback whale blubber. More specifically, the slopes of the linearized dilution curve of the blubber extracts (Plikaytis *et al*., 1994) were not significantly different from the assay standards (*P* = 0.121; Fig. 1). The pooled extracts had a slightly steeper slope than the progesterone standards [−0.81 vs. −0.77 logit (optical density)/log(relative dilution)]. Thus, at the low end of the detection range they measured slightly higher than the standards (2–3% maximal deflection in concentration; e.g. 8.0 pg/ml could measure as high as 8.2 pg/ml), and at the high end of the detection range they measured slightly lower than the progesterone standards (4–5% maximal deflection in concentration; e.g. 200 pg/ml could measure as high as 209 pg/ml). This variation in concentration between both dilution curves is within the manufacturer's reported inter-assay coefficient of variation (3–8%).

**Figure 2:**
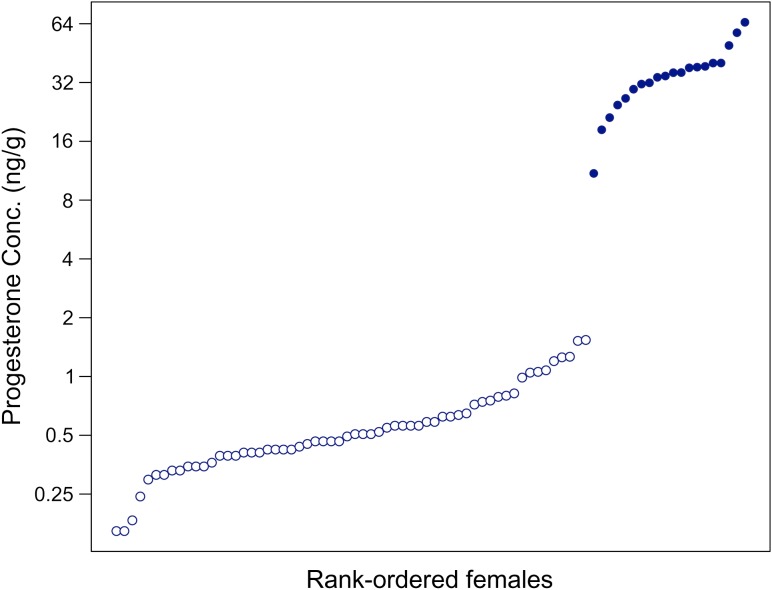
Blubber progesterone concentrations of rank-ordered female humpback whales. The vertical gap towards the right side of the plot illustrates the substantial difference between animals classified as pregnant (filled circles) and those classified as non-pregnant (open circles).

Blubber progesterone concentrations of female humpback whales exhibited a gap between 1.29 and 21.92 ng/g, with many values clustered below this gap and a smaller number distributed above to values as high as 286.53 ng/g (Fig. 2). This gap was consistent with the differences in progesterone concentrations observed by Mansour *et al*. (2002) among pregnant and non-pregnant minke whales. They reported a maximal value of 3.43 ng/g in non-pregnant females and a minimal value of 22.84 ng/g in pregnant animals. For the purposes of the present study, the gap in blubber progesterone was used as a threshold for diagnosing pregnancy. Animals with blubber progesterone concentrations below the gap were classified as non-pregnant (*n* = 38) and animals with progesterone values above the gap as pregnant (*n* = 20). Varying the progesterone concentration threshold above which animals were classified as pregnant had little effect on the results (Table 1).

**Table 2:** Blubber progesterone concentrations (in nanograms per gram) for male and female humpback whales of different maturity status, presented as means ± SEM

	Pregnant females	Mature females (non-pregnant)	Females (maturity unknown)	Mature males	Males (maturity unknown)
Mean	122.28	0.23	0.50	0.15	0.20
SEM	±15.07	±0.02	±0.06	±0.02	±0.03
Minimum	46.05	0.13	0.14	0.06	0.05
Maximum	286.53	0.32	1.29	0.22	0.46
*n*	20	8	30	6	16

Blubber progesterone values differed significantly among males, pregnant females and non-pregnant females (*F*_2,77_ = 116.2, *P* < 0.001). *Post hoc* analysis indicated that male humpbacks had significantly lower blubber progesterone concentrations than pregnant females (*P* < 0.001) but did not differ from non-pregnant females (*P* > 0.999). Mean (±SEM) blubber progesterone concentration was 122.28 ± 15.07 ng/g for pregnant females, 0.23 ± 0.02 ng/g for non-pregnant mature females and 0.50 ± 0.06 ng/g for non-pregnant females of unknown maturity status. Mean (±SEM) blubber progesterone concentration for mature male humpbacks was 0.15 ± 0.02 ng/g and for males of unknown maturity status 0.20 ± 0.03 ng/g (Table 2). The pregnancy rate of female humpback whales sampled in 2011 was 48.4% (*n* = 31) and was significantly greater than the pregnancy rate of 18.5% (*n* = 27) in 2012 (two-proportion *Z*-test: *Z* = 2.387, *P* = 0.017).

**Table 1:** Results of the test of the impact of varying the pregnancy criterion threshold on pregnancy diagnoses

Pregnancy criterion threshold (ng/g)	Females classified as pregnant (%)
5	34.48 (*n* = 20)
15	34.48 (*n* = 20)
25	32.76 (*n* = 19)
35	32.76 (*n* = 19)
45	32.76 (*n* = 19)
55	31.03 (*n* = 18)

Varying the pregnancy criterion threshold from 5 to 55 ng/g had a minimal effect on the results, changing the pregnancy classification of only two animals.

Progesterone analyses revealed an unexpected decline in pregnancy rate within the 2011 sampling period. Female whales sampled in the early/middle portions of the feeding season (May–July, *n* = 21) had a pregnancy rate of 63.6%, significantly greater (two-proportion *Z*-test: *Z* = 2.556, *P* =  0.010) than the 11.1% pregnancy rate of whales sampled in the late portion of the feeding season (October–November, *n* = 9). The shorter duration of sampling in 2012 (late April to early July) did not allow for investigation of changes in pregnancy rate within the 2012 feeding season. Blubber progesterone concentrations did not exhibit a significant linear relationship with day of year for pregnant females (*F*_1,13_ = 0.193, *P* = 0.668), non-pregnant females (*F*_1,14_ = 0.048, *P* = 0.831) or males (*F*_1,20_ = 0.111, *P* = 0.743).

**Figure 3:**
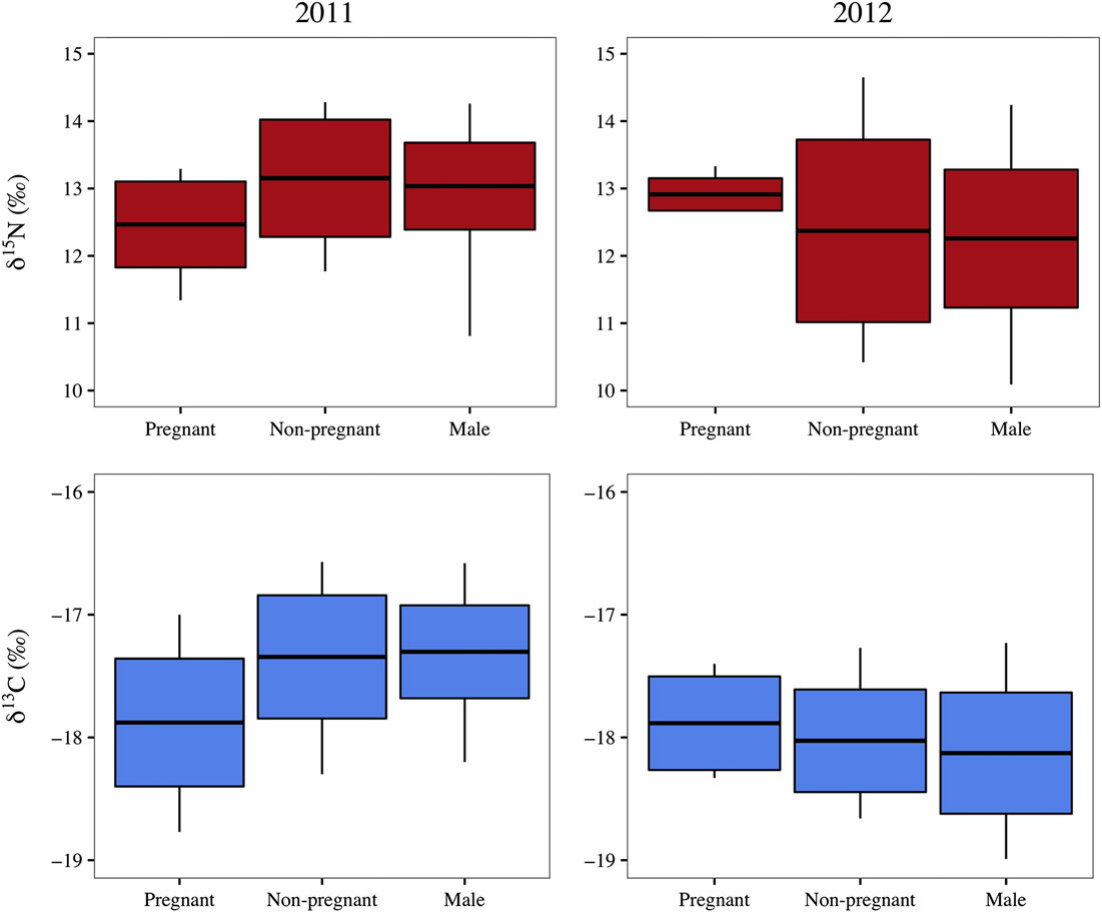
Stable carbon and nitrogen isotope ratios of male, non-pregnant female and pregnant female humpbacks. Mean δ^13^C (bottom) and δ^15^N (top) values for male, non-pregnant female and pregnant female humpbacks sampled in 2011 (left) and 2012 (right). The top and bottom of the boxplot represent one standard deviation away from the mean. Whiskers extend to the maximal and minimal values.

**Figure 4:**
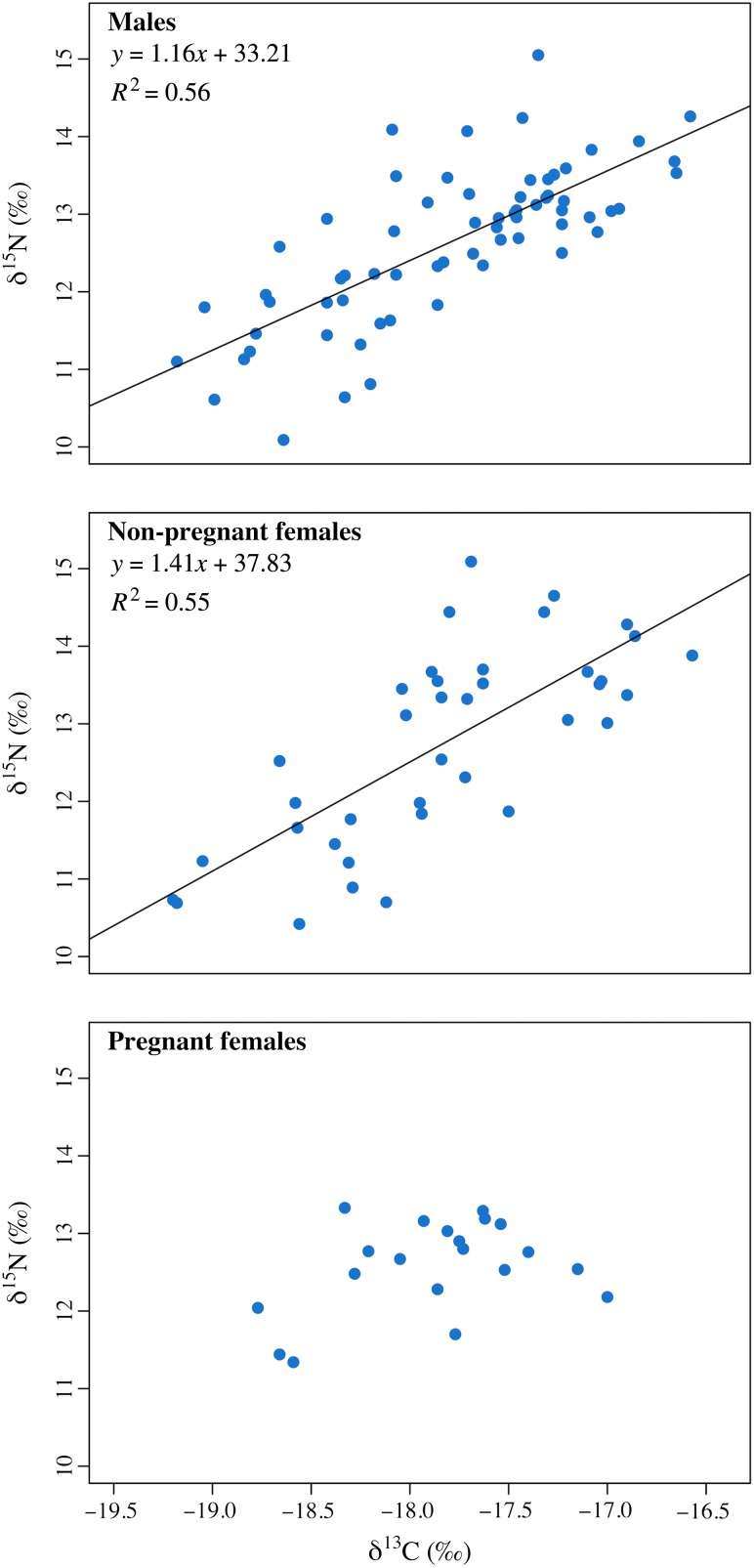
Relationships between δ^13^C and δ^15^N values for male, non-pregnant female and pregnant female humpback whales. Males and non-pregnant females exhibited a positive, linear relationship between δ^13^C and δ^15^N within individual animals, whereas pregnant females did not exhibit a relationship between these values.

In 2011, stable carbon and nitrogen isotope ratios differed significantly among animals of differing reproductive status (δ^13^C, *F*_2,57_ = 8.767, *P* < 0.001; δ^15^N, *F*_2,57_ = 4.88, *P* = 0.011). Tukey's HSD *post hoc* tests revealed that these differences were driven by pregnant females, which had significantly lower δ^13^C and δ^15^N values than other groups (*P* < 0.05), although the δ^15^N values of pregnant females and males did not differ significantly (*P* = 0.05). In 2012, however, there were no differences among animals of differing reproductive status for either δ^13^C or δ^15^N (δ^13^C, *F*_2,61_ = 0.724, *P* = 0.489; δ^15^N, *F*_2,61_ = 0.550, *P* = 0.580; Fig. 3). Male humpback whales exhibited a strong, positive relationship between δ^13^C and δ^15^N (*F*_1,64_ = 80.53, *P* < 0.001, *R*^2^ = 0.557), as did non-pregnant females (*F*_1,36_ = 43.38, *P* < 0.001, *R*^2^ = 0.547). In contrast, pregnant females did not exhibit a significant relationship (*F*_1,18_ = 3.050, *P* = 0.098, *R*^2^ = 0.145) between carbon and nitrogen stable isotope ratios (Fig. 4).

## Discussion

The results of the progesterone assays supported the use of this method as a tool to diagnose pregnancy in humpback whales and revealed previously unobserved trends in pregnancy rate, both within and between the years of this study. To be useful as a tool for classifying pregnancy status, the results of the progesterone assay needed to allow for clear differentiation between pregnant and non-pregnant animals. The mean concentration of blubber progesterone for whales classified as pregnant was two to three orders of magnitude greater than that of animals classified as non-pregnant. Differences in progesterone concentrations between these two groups were of a similar magnitude to those observed in other cetacean species (Mansour *et al*., 2002; Kellar *et al*., 2006; Pérez *et al*., 2011; Trego *et al*., 2013). Moreover, the distributions of blubber progesterone concentrations of whales sampled for this study were similar to those of pregnant, non-pregnant and male minke whales whose reproductive status was confirmed by direct analysis of reproductive organs (Mansour *et al*., 2002), leading us to conclude that this method was effective for diagnosing pregnancy in humpback whales.

Pregnancy rate varied substantially between the 2 years of this study (48.4% in 2011 and 18.5% in 2012); however, the magnitude of this variability was within the range of variation observed in other estimates of reproductive rates for humpback whales off California. Direct comparison of pregnancy rates with other estimates of reproduction is inherently problematic, as each estimate uses different metrics to quantify reproductive output. Calving rate is defined as the number of calves observed per mature female in a given year and is perhaps the most directly comparable measure. Steiger and Calambokidis (2000) reported an average calving rate of 0.44 for humpback whales off California, with a range of 0.14–0.73. The pregnancy rates presented here differ from those used in many wildlife studies (e.g. Derocher *et al*., 1992; Noyes *et al*., 1996) in that they do not take into account the maturity status of sampled whales. Thus, our estimates of pregnancy rate would be expected to be lower than the calving rate, as some of the females sampled for this study were likely to have been immature; however, factors such as fetal and perinatal mortality, as well as the decreased probability of sighting calves would be expected to inflate the pregnancy rate relative to the calving rate and might make these two measures of reproduction more directly comparable. Our estimates of pregnancy rate fell on either side of the estimate of 37.2% generated by Chittleborough (1965) from commercial whaling data.

On average, female humpback whales have a 2–3 year reproductive cycle, with 1 year devoted to pregnancy, 1 year for lactation and weaning and, for some whales, one resting year for body maintenance before subsequent reproduction (Chittleborough, 1965; Steiger and Calambokidis, 2000). For populations of animals with extended reproductive cycles, such as large whales, year-to-year variability in environmental conditions might be expected to result in some degree of synchronization in the timing of reproduction. For example, if a year of decreased prey availability (resulting in a disproportionate number of lost pregnancies) is followed by a year of greater prey availability (resulting in a disproportionate number of successful pregnancies), reproductive output during the second year would be increased, as both females with unsuccessful pregnancies the previous year and females that normally would have reproduced during the second year would probably become pregnant. The result would be a degree of inter-annual synchronicity in reproductive output, which might explain the variability in pregnancy and calving rates observed in this study and other investigations of large whale reproduction (Steiger and Calambokidis, 2000; Kraus *et al*., 2001). This phenomenon has been observed in other cetaceans (Whitehead and Mann, 2000), but has not been the subject of much research. Synchronization of reproduction may occur for a number of reasons, including ‘dilution’ of predation risk for young calves attributable to the presence of other, similar-sized calves, reduction of competition among cohorts of offspring, in addition to environmental forcing (Whitehead and Mann, 2000). Further research is needed to determine which of these factors is most important in driving variability in reproductive output of large whale populations.

The steep decrease in pregnancy rate between the early and late portions of the 2011 field season was unexpected and raises questions regarding the reproductive biology of humpback whales. Although it is possible that the observed trend was the result of small sample sizes, the magnitude of the difference between the early/middle portions of the feeding season and the late feeding season supports the idea that these results represent a real effect. It is unlikely that the decline in pregnancy rate would result from false positives among animals classified as pregnant early in the year. The magnitude of the difference between the pregnant female with the lowest progesterone concentration and the non-pregnant female with the highest progesterone concentration was substantially greater than seasonal changes in hormone concentrations typical of other cetaceans (Atkinson *et al*., 1999; Robeck *et al*., 2009). Differences between progesterone concentrations of non-pregnant and pregnant animals in this study were at least an order of magnitude greater than seasonal fluctuations in captive cetaceans. Furthermore, shifts in blubber progesterone concentration associated with changes in reproductive status typically occur over short time spans in cetaceans, of the order of tens of hours or days (Kellar *et al*., 2006). Humpback whale reproduction peaks in February and March (Au *et al*., 2000), and the fastest documented migration for this species was 39 days (Gabriele and Straley, 1996), thus these changes would probably have occurred before the animals reached Californian waters.

Another explanation for the observed change in pregnancy rate is a shift in habitat use or decrease in the likelihood of sampling pregnant whales. If pregnant females tend to exploit a particular type of habitat or exhibit migratory behaviours that differ from other demographic groups, it is possible that changes in pregnancy rate in this study reflect movements by these whales in and out of the study area, which represents only a small portion of the feeding area off California and Oregon. There is evidence that humpback whales use different habitats based on sex, age and reproductive status on a small scale in breeding areas (Smultea, 1994; Craig and Herman, 2000; Ersts and Rosenbaum, 2003). Within feeding areas, female humpbacks with dependent calves may use slightly different habitats from other whales (Steiger and Calambokidis, 2000), but otherwise, habitat stratification or segregation has not been reported for this species (Robbins, 2007). Biopsy collection itself may be subject to a number of sampling biases, including underrepresentation of animals that spend less time at the surface, are difficult to see or actively avoid the research vessel. Conversely, animals that spend more time at the surface, are more easily sighted or are less likely to avoid the sampling platform may be overrepresented in the sample. If females with calves were not present in the study area in large numbers or if they actively avoided the vessel from which sampling was conducted, this might have contributed to the high pregnancy rate early in the 2011 feeding season, as many non-pregnant females would have been unavailable for sampling. The likelihood of sampling these females may then have increased later in the year with the weaning of their dependent calves, contributing to the low pregnancy rate at this time.

Finally, it is possible that the observed changes in pregnancy rate reflect a genuine decrease in the number of pregnant animals across the 2011 feeding season. Some animal species exhibit embryonic/fetal resorption or early termination of pregnancy during periods of stress, including disease and nutritional stress (Conaway *et al*., 1960; Huck *et al*., 1988). It is possible that the observed decrease in pregnancy rate across the 2011 feeding season resulted from embryonic/fetal resorption or termination of pregnancy triggered by an unknown stressor or stressors. Hailey *et al*. (1966) suggested that fetal resorption may be an adaptive mechanism that allows pregnant females to respond to changing environmental conditions. Such a mechanism would allow pregnant humpback whales to respond to unpredictable oceanographic conditions, maximizing reproductive output in years when conditions were favourable and incurring only the minimal costs associated with the early stages of pregnancy in unfavourable years. This might help to explain the variable reproductive output of the population among years; however, this phenomenon has not yet been reported for marine mammals. Alternatively, the decrease in pregnancy rate might have resulted from pregnant females beginning the migration to the breeding grounds earlier than non-pregnant whales. Pregnant humpbacks and females with new calves are the last groups to arrive in the breeding areas (Craig *et al*., 2003); however, these animals probably travel the slowest because they are either in the late stages of pregnancy or are accompanied by a calf born *en route*. Non-pregnant females may benefit from staying in feeding areas for extended periods of time, perhaps even overwintering there, to improve body condition in preparation for another reproductive cycle. Each of these hypotheses merits further investigation.

**Figure 5:**
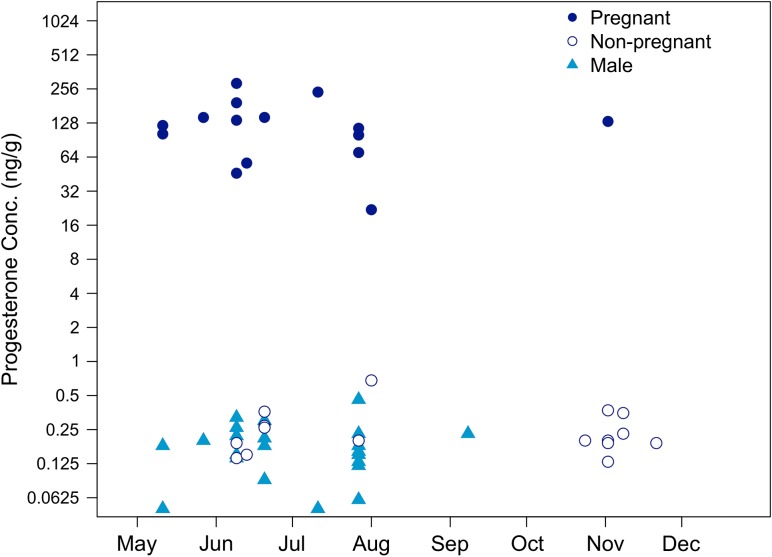
Progesterone concentrations (in nanograms per gram) of pregnant (filled circles), non-pregnant female (open circles) and male (filled triangles) humpback whales sampled in 2011 plotted by sampling date. No significant relationships existed between progesterone concentration and sampling date for any group.

We did not find evidence of seasonal fluctuations in blubber progesterone concentrations for male or non-pregnant female humpbacks (Fig. 5). Captive false killer whales (*Pseudorca crassidens*) and Pacific white-sided dolphins (*Lagenorhynchus obliquidens*) both exhibit elevated progesterone concentrations for extended periods during and subsequent to ovulation (Atkinson *et al*., 1999; Robeck *et al*., 2009), lasting for as long as 103 days in the white-sided dolphin. Although it is an important caveat that comparisons between captive and wild animals are problematic because of the conditions of captivity (i.e. consistent diet and water temperature, inability to migrate, etc.), the absence of seasonal changes in the blubber progesterone concentrations of female humpback whales sampled in this study may indicate that females do not ovulate annually or that the increased production of progesterone associated with ovulation had diminished by the time the animals reached Californian waters. Studies of reproductive tracts of humpback whales killed by commercial whalers indicated an average ovulation rate of slightly greater than once per year (Chittleborough, 1965), leading us to conclude that the second explanation is more likely to be correct. Likewise, the progesterone concentrations of pregnant females were relatively stable across the feeding season. Although it would be ideal to obtain serial samples from individual whales to quantify any changes in progesterone concentrations that occur during pregnancy, longitudinal sampling of the population would probably be sufficient to capture any large-scale increase or decline in progesterone concentration through time. Our inference here is limited by the inclusion of only a single pregnant female sampled late in the year; however, blubber progesterone concentrations of pregnant humpbacks remained generally constant during the early/middle portion of the feeding season and, although it certainly cannot be considered a representative sample, the single data point from the late portion of the feeding season was roughly equivalent to those from individuals sampled earlier in the year (Fig. 5).

Investigations of trends in progesterone concentrations for other animals have produced highly variable results (e.g. Ranilla *et al*., 1994; Sousa *et al*., 1999); however, the relatively stable progesterone concentrations presented here fit well with the results of other studies of cetacean hormone concentrations during pregnancy. Bergfelt *et al*. (2011) reported no change in the serum progesterone concentrations of bottlenose dolphins (*Tursiops truncatus*) in the early and middle periods of pregnancy, followed by a decline in late pregnancy. Likewise, Hunt *et al*. (2014) reported progesterone concentrations in the baleen of bowhead whales that remained relatively stable during pregnancy, then dropped sharply at or near parturition. Robeck *et al*. (2016) observed a peak in killer whale (*Orcinus orca*) serum progesterone values shortly after the initiation of pregnancy, followed by a small decline, then relatively stable values until shortly before parturition. Direct comparison with these studies is complicated by the analysis of different tissue types, which are likely to experience changes in hormone concentrations at different rates; however, the general trend exhibited by the humpback whale blubber progesterone concentrations is similar to trends observed in the tissues of other cetaceans. The relationship between hormone concentrations in blubber and other tissues, such as plasma, baleen and mucosa from blow, merits further study, and serial sampling of known individuals in a well-studied population (e.g. Gulf of Maine humpback whales) would provide a much better understanding of changes in hormone concentrations during pregnancy.

In addition to allowing investigations into endocrinology and demography, the ability to assign pregnancy status to individuals opens up a variety of research opportunities related to the reproduction of wild animals. Stable isotope analysis is a particularly compelling avenue of study as it can provide information about both the physiology and the ecology of an animal, requires only a small amount of tissue and is relatively inexpensive. This is a convenient method for use with cetacean biopsy samples, as only the blubber is used to measure progesterone concentrations, leaving the remainder of the sample available for other analyses. Previous research on humpback whales in this region in 2011 and 2012 found little intra-annual variation in diet among individuals and no sex-related differences in stable carbon and nitrogen isotope ratios (Fleming *et al*., 2015). Thus, diet-related differences can be largely discounted when comparing isotope ratios of humpbacks within each year of this study.

The impacts of pregnancy on stable isotope ratios within the bodies of female animals have historically been understudied. For marine mammals, this work has been restricted primarily to pinnipeds (e.g. Kurle and Worthy, 2001; Habran *et al*., 2010), which typically breed on land and for which reproductive status can often be determined by observation. Hunt *et al*. (2016) examined hormone concentrations in bowhead baleen and used fluctuations in stable isotope ratios along the baleen plates to establish annual growth rates, but did not consider the links between the two signals. To our knowledge, this is the first time that the relationship between pregnancy status and stable isotope ratios has been investigated in tandem for a cetacean species. Such research may provide insight into the physiological and energetic demands of pregnancy and may hold important implications for research that uses stable isotopes as a tool for addressing questions about animal physiology, ecology and life history.

**Figure 6:**
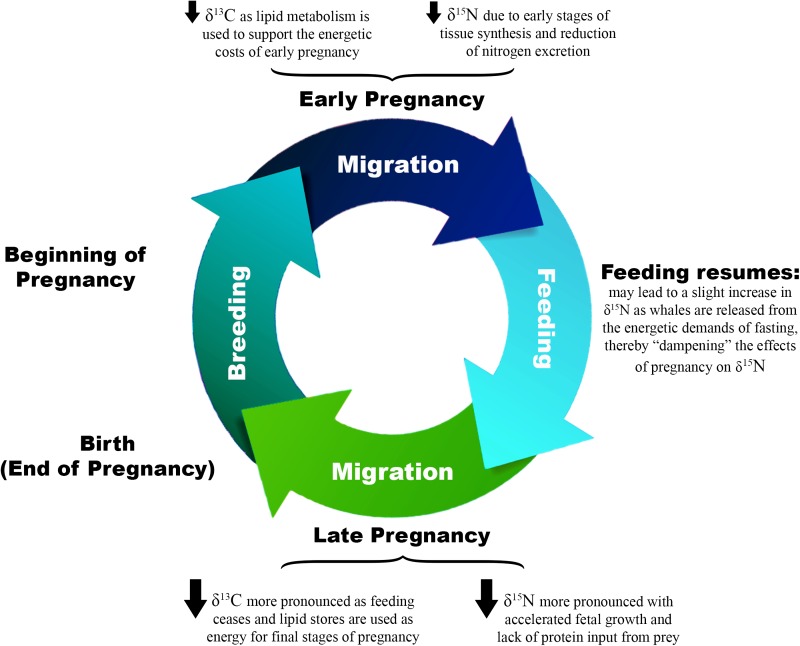
Proposed theoretical framework for hypothesized changes in δ^13^C and δ^15^N of pregnant humpback whales relative to non-pregnant animals throughout pregnancy. This diagram describes the proposed mechanisms through which pregnancy impacts δ^13^C and δ^15^N, including the metabolism of blubber lipid stores, tissue synthesis, reduced nitrogen excretion and seasonal fasting. One full revolution of the proposed cycle begins with conception during the breeding period and ends with birth at the beginning of the subsequent breeding period. Lactation would be expected to generate further changes in the isotope ratios of both mother and calf; however, this is not discussed here. The size of the arrows indicates the hypothesized magnitude of the changes in δ^13^C and δ^15^N. This theoretical model does not consider differences in diet among individuals or at different stages of pregnancy. References: Kelly (2000); Kurle and Worthy (2001); Fuller *et al*. (2004); Martínez Del Rio *et al*. (2009); Newsome *et al*. (2010); Kurle *et al*. (2014).

We propose the following theoretical framework to describe the physiological and isotopic changes that occur during the reproductive cycle of this migratory species (Fig. 6). During pregnancy, protein synthesis increases, a process which decreases nitrogen excretion and consequently isotopic fractionation, by retaining more ^14^N than animals in stasis (Fuller *et al*., 2004; Martínez Del Rio *et al*., 2009; Kurle *et al*., 2014). As a result of these physiological changes, the δ^15^N values of pregnant animals would be expected to be lowered relative to other animals (Newsome *et al*., 2010). Simultaneously, utilization of lipid stores to meet the energetic demands of pregnancy, especially during periods of migration, probably cause δ^13^C values to decrease, as lipids are isotopically light in comparison to proteins (Kelly, 2000; Kurle and Worthy, 2001). In a species like the humpback whale that is adapted to extended periods of fasting and migration, blubber stores are likely to be the primary source of energy until stages of extreme nutritional stress are reached (Aguilar *et al*., 2014).

Given this theoretical framework, we would expect pregnant female humpbacks sampled for this study to have lower δ^13^C and δ^15^N values than both non-pregnant female and male whales. Our results indicated that this was true for animals sampled in 2011; however, in 2012 there were no differences among these groups. Unfortunately, the strength of these analyses was diminished by the fact that only one of the whales sampled late in the year in 2011 was pregnant, greatly reducing our ability to draw conclusions about this period. To ensure strong inference and to allow for more direct comparisons among years, we reanalysed the 2011 data from the portion of the year most closely approximating the 2012 sampling period (May–July). When analysis of the 2011 samples was restricted to this period, the differences in δ^15^N values between pregnant females and both non-pregnant and female whales were no longer significant (*F*_2,27_ = 1.456, *P* = 0.251). The δ^13^C values still differed significantly by reproductive status (*F*_2,27_ = 8.359, *P* = 0.001); however, a Tukey's HSD *post hoc test* indicated that this result was driven entirely by the difference between pregnant females and males (*P* = 0.001). Non-pregnant females no longer differed from pregnant whales (*P* = 0.056). Multiple factors may be responsible for the similar isotope ratios of pregnant females and other animals in the early/middle portions of the feeding season in both years of this study. First, it may be that in the early stages of pregnancy, protein synthesis is still minimal, thus the effects of tissue deposition and decreased excretion may not yet be detectable. Second, it is important to consider that the stable isotope ratios of humpback skin reflect the physiology/diet of the whale at the time when the tissue was generated. The turnover rate of humpback whale skin has not been measured, but it has been estimated to be ~7–14 days (Todd, 1997). Hicks *et al*. (1985) measured a longer turnover rate of ~73 days for bottlenose dolphin skin cells. Thus, it can be assumed that humpback whale skin represents a period of time ranging from weeks to months prior to sampling. For the purposes of our study, this would mean that the isotope ratios in the skin of pregnant females reflected an earlier stage of pregnancy when the effects on δ^13^C and δ^15^N were less pronounced. Finally, because we sampled in the feeding area, the individuals sampled for our study were likely to have resumed feeding and may therefore have been using new protein sources from their diet. This might result in a return to more a more typical nitrogen excretion regime, thereby increasing their δ^15^N and dampening the protein synthesis signal.

What little research has been done on this topic offers additional support for this proposal. A study of stable nitrogen isotopes in human hair found the expected decline in δ^15^N values during pregnancy and no changes in δ^13^C values (Fuller *et al*., 2004). The lack of change in δ^13^C is probably explained by the absence of extensive fat stores or fasting behaviour in humans. Whereas whales almost certainly rely on their large lipid reserves during various stages of pregnancy, humans maintain a regular diet throughout pregnancy and would not be expected to experience a decline in δ^13^C. A study of polar bears found mixed results, with three study groups experiencing a decrease in δ^15^N during pregnancy and two experiencing an increase (Polischuk *et al*., 2001); however, Polischuk *et al*. (2001) attributed the disparity among these groups to the body condition of the mothers. The females that experienced a drop in δ^15^N had ample fat reserves and were less likely to have metabolized ^15^N-enriched proteins during fasting (Polischuk *et al*., 2001). Likewise, in humpback whales, the body condition of the mother is likely to be a factor in determining the likelihood of pregnancy, and consequently, our likelihood of detecting early- and mid-stage pregnancies in the Monterey Bay feeding area. Individuals with larger blubber reserves and better body condition may be more likely to become pregnant and retain that pregnancy until the time at which they were sampled. This superior body condition would be likely to result in relatively less protein metabolism during the preceding migratory cycle and thereby a lower δ^15^N signature upon returning to the feeding area.

As a group, humpback whales sampled for this study exhibited a strongly positive, linear relationship between δ^13^C and δ^15^N values. The relationship between δ^13^C and δ^15^N is likely to reflect nutrient utilization by phytoplankton at the base of the food web and would therefore be expected to be consistent across demographic groups of humpback whales feeding simultaneously in the same region (Waser *et al*., 1998). When the demographic groups were looked at individually, this relationship was exhibited by male and non-pregnant female humpbacks, but not by pregnant female whales. Thus, despite there being few differences among the mean δ^15^N and δ^13^C values of the different demographic groups (particularly in the early/middle portion of the feeding season), the relationship between these two ratios within individual animals was substantially different in the skin of pregnant females from that in other whales. The decoupling of this relationship, as opposed to a change in the slope or *y*-intercept of the regression line, indicates that pregnancy affected the stable isotope ratios of pregnant females at varying magnitudes and/or with varying timing among individuals. It is unclear whether this decoupling occurred as a result of changes in δ^15^N or δ^13^C values (or both). The conclusions drawn by Fuller *et al*. (2004) may provide an explanation of our results. They observed a decline in δ^15^N values of human hair over the course of pregnancy; however, this decline was not linear. Rather, δ^15^N values declined more rapidly during periods when the mothers gained weight. Additionally, changes in maternal δ^15^N values from conception to birth were correlated with the birth weight of the infant. Thus, the magnitude and timing of changes in the stable isotope ratios of pregnant females sampled in our study might be expected to differ substantially among whales based on individual variation in fetal growth and the timing of pregnancy, and could account for the decoupling of the relationship between δ^15^N and δ^13^C values we observed. This conclusion has particularly important implications for studies that use stable isotope ratios to draw conclusions about foraging behaviour, as it provides further support for the concept that the stable isotope ratios of pregnant females do not exclusively reflect diet. Although it is not feasible for every diet study to incorporate pregnancy testing, further research into the timing and magnitude of pregnancy-related changes in stable isotope ratios, as well as the proportion of pregnant animals in the population in a given year (i.e. average pregnancy rate), may help to improve future stable isotope studies and may shed light on the energetic demands of reproduction in species that are difficult to study.

### Conclusions

Progesterone assays of blubber appear to be an effective tool for diagnosing pregnancy in female humpback whales. The pregnancy rate estimates we generated using this technique differed between the 2 years of this study, but fell within the range of previous estimates of calving rates for whales off California. Our estimates of pregnancy rate were generated from relatively small sample sizes and were restricted to a small geographical area. Future work encompassing a broader geographical range and sampling a larger number of individuals would probably create a pregnancy rate estimate that is more representative of the humpback whale feeding aggregation that forages off California and Oregon. Our results suggest that it is also valuable to sample across multiple years, as the pregnancy rate varies from year to year, highlighting the importance of longitudinal studies. We propose a working hypothesis for the impacts of pregnancy on stable isotopes, suggesting that both δ^15^N and δ^13^C decline during pregnancy, due mainly to protein synthesis and lipid metabolism. The decoupling of the relationship between δ^13^C and δ^15^N values in these animals suggests that the magnitude and timing of these impacts varies among individuals and with stage of pregnancy. Thus, unless they are better understood, these pregnancy-related changes to the stable isotope ratios have the potential to confound studies that use isotopes to investigate topics such as diet and migration. Taken together, the results of this study provide an important tool for better understanding the reproductive biology and physiology of cetaceans, which will assist monitoring, management and conservation efforts in an uncertain future.

## References

[cow050C1] Aguilar A, Giménez J, Gómez-Campos E, Cardona L, Borrell A (2014) δ^15^N value does not reflect fasting in mysticetes. PLoS One 9: e92288. 24651388 10.1371/journal.pone.0092288 PMC3961314

[cow050C2] Atkinson S, Combelles C, Vincent D, Nachtigall P, Pawloski J, Breese M (1999) Monitoring of progesterone in captive female false killer whales, *Pseudorca crassidens*. Gen Comp Endocrinol 115: 323–332. 10480983 10.1006/gcen.1999.7319

[cow050C3] Au WWL, Mobley JR, Burgess W, Lammers MO, Nachtigall PE (2000) Seasonal and diurnal trends of chorusing humpback whales wintering in waters off Western Maui. Mar Mammal Sci 16: 530–544.

[cow050C4] Bergfelt DR, Steinetz BG, Lasano S, West KL, Campbell M, Adams GP (2011) Relaxin and progesterone during pregnancy and the post-partum period in association with live and stillborn calves in bottlenose dolphins (*Tursiops truncatus*). Gen Comp Endocrinol 170: 650–656. 21156178 10.1016/j.ygcen.2010.12.002

[cow050C5] Chittleborough R (1965) Dynamics of two populations of the humpback whale *Megaptera novaeangliae* (Borowski). Aust J Mar Freshw Res 1964: 33–128.

[cow050C6] Conaway CH, Baskett TS, Toll JE (1960) Embryo resorption in the swamp rabbit. J Wildl Manage 24: 197–202.

[cow050C7] Craig AS, Herman LM (2000) Habitat preferences of female humpback whales *Megaptera novaeangliae* in the Hawaiian Islands are associated with reproductive status. Mar Ecol Prog Ser 193: 209–216.

[cow050C8] Craig AS, Herman LM, Gabriele CM, Pack AA (2003) Migratory timing of humpback whales (*Megaptera novaeangliae*) in the central North Pacific varies with age, sex and reproductive status. Behaviour 140: 981–1001.

[cow050C9] Derocher AE, Stirling I, Andriashek D (1992) Pregnancy rates and serum progesterone levels of Polar bears in western Hudson Bay. Can J Zool 70: 561–566.

[cow050C10] Ersts PJ, Rosenbaum HC (2003) Habitat preference reflects social organization of humpback whales (*Megaptera novaeangliae*) on a wintering ground. J Zool 260: 337–345.

[cow050C11] Fleming AH, Clark CT, Calambokidis J, Barlow J (2015) Humpback whale diets respond to variance in ocean climate and ecosystem conditions in the California Current. Glob Chang Biol 22: 1214–1224. 26599719 10.1111/gcb.13171

[cow050C12] Fuller BT, Fuller JL, Sage NE, Harris DA, O'Connell TC, Hedges REM (2004) Nitrogen balance and δ15N: why you're not what you eat during pregnancy. Rapid Commun Mass Spectrom 18: 2889–2896. 15517531 10.1002/rcm.1708

[cow050C13] Gabriele C, Straley JM (1996) Fastest documented migration of a North Pacific humpback whale. Mar Mammal Sci 12: 457–464.

[cow050C14] Gilson A, Syvanen M, Levine K, Banks J (1998) Deer gender determination by poymerase chain reaction: validation study and application to tissues, bloodstains, and hair from forensic samples from California. Calif Fish Game 84: 159–169.

[cow050C15] Habran S, Debier C, Crocker DE, Houser DS, Lepoint G, Bouquegneau JM, Das K (2010) Assessment of gestation, lactation and fasting on stable isotope ratios in northern elephant seals (*Mirounga angustirostris*). Mar Mammal Sci 26: 880–895.

[cow050C16] Hailey T, Thomas J, Robinson R (1966) Pronghorn die-off in Trans-Pecos Texas. J Wildl Manage 30: 488–496.

[cow050C17] Hicks BD, St Aubin DJ, Geraci JR, Brown WR (1985) Epidermal growth in the bottlenose dolphin, *Tursiops truncatus*. J Invest Dermatol 85: 60–63. 4008976 10.1111/1523-1747.ep12275348

[cow050C18] Hobson KA (1999) Tracing origins and migration of wildlife using stable isotopes: a review. Oecologia 120: 314–326. 28308009 10.1007/s004420050865

[cow050C19] Huck WU, Lisk RD, Miller KS, Bethel A (1988) progesterone levels and socially-induced implantation failure and fetal resorption in golden hamsters (*Mesocricetus auratus*). Physiol Behav 44: 321–326. 3065803 10.1016/0031-9384(88)90032-7

[cow050C20] Hunt KE, Stimmelmayr R, George C, Hanns C, Suydam R, Brower H, Rolland RM (2014) Baleen hormones: a novel tool for retrospective assessment of stress and reproduction in bowhead whales (*Balaena mysticetus*). Conserv Physiol 2: cou030; doi:10.1093/conphys/cou030. 27293651 10.1093/conphys/cou030 PMC4806734

[cow050C21] Hunt KE, Lysiak NS, Moore MJ, Rolland RM (2016) Longitudinal progesterone profiles in baleen from female North Atlantic right whales (*Eubalaena glacialis*) match known calving history. Conserv Physiol 4: cow014; doi:10.1093/conphys/cow014. 27293762 10.1093/conphys/cow014 PMC4864594

[cow050C22] Kellar NM, Trego ML, Marks CI, Dizon AE (2006) Determining pregnancy from blubber in three species of delphinids. Mar Mammal Sci 22: 1–16.

[cow050C23] Kellar NM, Keliher J, Trego ML, Catelani KN, Hanns C, George JCC, Rosa C (2013a) Variation of bowhead whale progesterone concentrations across demographic groups and sample matrices. Endanger Species Res 22: 61–72.

[cow050C24] Kellar NM, Trego ML, Chivers SJ, Archer FI (2013b) Pregnancy patterns of pantropical spotted dolphins (*Stenella attenuata*) in the eastern tropical Pacific determined from hormonal analysis of blubber biopsies and correlations with the purse-seine tuna fishery. Mar Biol 160: 3113–3124.

[cow050C25] Kellar NM, Trego ML, Chivers SJ, Archer FI, Perryman WL (2014) From progesterone in biopsies to estimates of pregnancy rates: large scale reproductive patterns of two sympatric species of common dolphin, *Delphinus* spp. off California, USA and Baja, Mexico. Bull South Calif Acad Sci 113: 58–80.

[cow050C26] Kellar NM, Catelani KN, Robbins MN, Trego ML, Allen CD, Danil K, Chivers SJ (2015) Blubber cortisol: a potential tool for assessing stress response in free-ranging dolphins without effects due to sampling. PLoS One 10: e0115257. 25643144 10.1371/journal.pone.0115257 PMC4314064

[cow050C27] Kelly JF (2000) Stable isotopes of carbon and nitrogen in the study of avian and mammalian trophic ecology. Can J Zool 78: 1–27.

[cow050C28] Kraus SD, Hamilton PK, Kenney RD, Knowlton AR, Slay CK (2001) Reproductive parameters of the North Atlantic right whale. J Cetacean Res Manag 2: 231–236.

[cow050C29] Kurle CM (2002) Stable-isotope ratios of blood components from captive northern fur seals (*Callorhinus ursinus*) and their diet: applications for studying the foraging ecology of wild otariids. Can J Zool 80: 902–909.

[cow050C30] Kurle CM, Worthy GAJ (2001) Stable isotope assessment of temporal and geographic differences in feeding ecology of northern fur seals (*Callorhinus ursinus*) and their prey. Oecologia 126: 254–265. 28547625 10.1007/s004420000518

[cow050C31] Kurle CM, Koch PL, Tershy BR, Croll DA (2014) The effects of sex, tissue type, and dietary components on stable isotope discrimination factors (Δ^13^C and Δ^15^N) in mammalian omnivores. Isotopes Environ Health Stud 50: 307–321. 24787278 10.1080/10256016.2014.908872

[cow050C32] Lambertsen RH (1987) A biopsy system for large whales and its use for cytogenetics. J Mammal 68: 443–445.

[cow050C33] Lasley BL, Kirkpatrick JF (1991) Monitoring ovarian function in captive and free-ranging wildlife by means of urinary and fecal steroids. J Zoo Wildl Med 22: 23–31.

[cow050C34] Mansour AA, Mkay DW, Lien J, Orr JC, Banoub JH, Ølen N, Stenson G (2002) Determination of pregnancy status from blubber samples in minke whales (*Balaenoptera acutorostrata*). Mar Mammal Sci 18: 112–120.

[cow050C35] Martínez Del Rio C, Wolf N, Carleton SA, Gannes LZ (2009) Isotopic ecology ten years after a call for more laboratory experiments. Biol Rev 84: 91–111. 19046398 10.1111/j.1469-185X.2008.00064.x

[cow050C36] Newsome SD, Clementz MT, Koch PL (2010) Using stable isotope biogeochemistry to study marine mammal ecology. Mar Mammal Sci 26: 509–572.

[cow050C37] Noyes JH, Johnson BK, Bryant LD, Findholt SL, Thomas JW (1996) Effects of bull age on conception dates and pregnancy rates of cow elk. J Wildl Manage 60: 508–517.

[cow050C38] Pérez S, García-López Á, De Stephanis R, Giménez J, García-Tiscar S, Verborgh P, Mancera JM, Martínez-Rodriguez G (2011) Use of blubber levels of progesterone to determine pregnancy in free-ranging live cetaceans. Mar Biol 158: 1677–1680.

[cow050C39] Plikaytis BD, Holder PF, Pais LB, Maslanka SE, Gheesling LL, Carlone GM (1994) Determination of parallelism and nonparallelism in bioassay dilution curves. J Clin Microbiol 32: 2441–2447. 7814480 10.1128/jcm.32.10.2441-2447.1994 PMC264081

[cow050C40] Polischuk SC, Hobson KA, Ramsay MA (2001) Use of stable-carbon and -nitrogen isotopes to assess weaning and fasting in female polar bears and their cubs. Can J Zool 79: 499–511.

[cow050C41] Ranilla M, Sulon J, Carro M, Mantecon A, Beckers J (1994) Plasmatic profiles of pregnancy-associated glycoprotein and progesterone levels during gestation in Churra and Merino sheep. Theriogenology 42: 537–545. 16727560 10.1016/0093-691x(94)90691-b

[cow050C42] R Core Team (2016) R: A language and environment for statistical computing. R Foundation for Statistical Computing, Vienna, Austria. https://www.R-project.org/.

[cow050C43] Robbins J (2007) Structure and dynamics of the gulf of maine humpback whale population. PhD Thesis: University of St Andrews.

[cow050C44] Robeck TR, Steinman KJ, Greenwell M, Ramirez K, Van Bonn W, Yoshioka M, Katsumata E, Dalton L, Osborn S, O'Brien JK (2009) Seasonality, estrous cycle characterization, estrus synchronization, semen cryopreservation, and artificial insemination in the Pacific white-sided dolphin (*Lagenorhynchus obliquidens*). Reproduction 138: 391–405. 19494046 10.1530/REP-08-0528

[cow050C45] Robeck TR, Steinman KJ, Brien JKO (2016) Characterization and longitudinal monitoring of serum progestagens and estrogens during normal pregnancy in the killer whale (*Orcinus orca*). Gen Comp Endocrinol 236: 83–97. 27401258 10.1016/j.ygcen.2016.07.010

[cow050C46] RStudio Team (2015) RStudio: Integrated Development for R. RStudio, Inc., Boston, MA http://www.rstudio.com/.

[cow050C47] Smultea MA (1994) Segregation by humpback whale (*Megaptera novaeangliae*) cows with a calf in coastal habitat near the island of Hawaii. Can J Zool 72: 805–811.

[cow050C48] Sousa NM, Garbayo JM, Figueiredo JR, Sulon J, Goncalves P, Beckers JF (1999) Pregnancy-associated glycoprotein and progesterone profiles during pregnancy and postpartum in native goats from the north-east of Brazil. Small Rumin Res 32: 137–147.

[cow050C49] Steiger GH, Calambokidis J (2000) Reproductive rates of humpback whales off Caifornia. Mar Mammal Sci 16: 220–239.

[cow050C50] Todd S (1997) Dietary patterns of humpback whales (*Megaptera novaeangliae*) in the Northwest Atlantic: evidence from ^13^C and ^15^N stable isotopes. Doctoral (PhD) thesis. Memorial University of Newfoundland.

[cow050C51] Trego ML, Kellar NM, Danil K (2013) Validation of blubber progesterone concentrations for pregnancy determination in three dolphin species and a porpoise. PLoS One 8: e69709. 23936083 10.1371/journal.pone.0069709 PMC3728358

[cow050C52] Vu ET, Clark C, Catelani K, Kellar NM, Calambokidis J (2015) Seasonal blubber testosterone concentrations of male humpback whales (*Megaptera novaeangliae*). Mar Mammal Sci 31: 1258–1264.

[cow050C53] Waser NAD, Harrison PJ, Nielsen B, Calvert SE, Turpin DH (1998) Nitrogen isotope fractionation during the uptake and assimilation of nitrate, nitrite, ammonium, and urea by a marine diatom. Limnol Oceanogr 43: 215–224.

[cow050C54] Whitehead H, Mann J (2000) Female reproductive strategies of cetaceans. In J Mann, PL Tyack, H Whitehead, H, eds, Cetacean Societies: Field Studies of Dolphins and Whales. University of Chicago Press, Chicago, IL, USA, pp 219–246.

[cow050C55] Whitten K, Garner G, Mauer F, Harris R (1992) Productivity and early calf survival in the Porcupine caribou herd. J Wildl Manage 56: 201–212.

