# Heavy with child? Pregnancy status and stable isotope ratios as determined from biopsies of humpback whales

**DOI:** 10.1093/conphys/cox047

**Published:** 2017-08-02

**Authors:** Casey T. Clark, Alyson H. Fleming, John Calambokidis, Nicholas M. Kellar, Camryn D. Allen, Krista N. Catelani, Michelle Robbins, Nicole E. Beaulieu, Debbie Steel, James T. Harvey

*Conserv Physiol* (2016) 4 (1): cow050; doi:10.1093/conphys/cow050

In the original version of this article, the publisher has erroneously tagged the article with a first and last page value. The correct citation for this paper should be Conserv Physiol (2016) 4 (1): cow050.

The publisher apologises for this error.

