# Corrigendum to: Heavy with child? Pregnancy status and stable isotope ratios as determined from biopsies of humpback whales

**DOI:** 10.1093/conphys/coz013

**Published:** 2019-05-10

**Authors:** Casey T Clark, Alyson H Fleming, John Calambokidis, Nicholas M Kellar, Camryn D Allen, Krista N Catelani, Michelle Robbins, Nicole E Beaulieu, Debbie Steel, James T Harvey

**Affiliations:** 1 Moss Landing Marine Laboratories, Moss Landing, CA, USA; 2 College of Fisheries and Ocean Sciences, University of Alaska Fairbanks, Fairbanks, AK, USA; 3 Department of Paleobiology and Vertebrate Zoology, National Museum of Natural History Smithsonian Institution, Washington, DC, USA; 4 Cascadia Research Collective, Olympia, WA, USA; 5 Marine Mammal and Turtle Division, Southwest Fisheries Science Center, National Marine Fisheries Service, National Oceanic and Atmospheric Administration, La Jolla, CA, USA

Figure 2 of this paper has been amended to correct a formatting error.

The corrected figure is displayed below.

**Figure 2:**
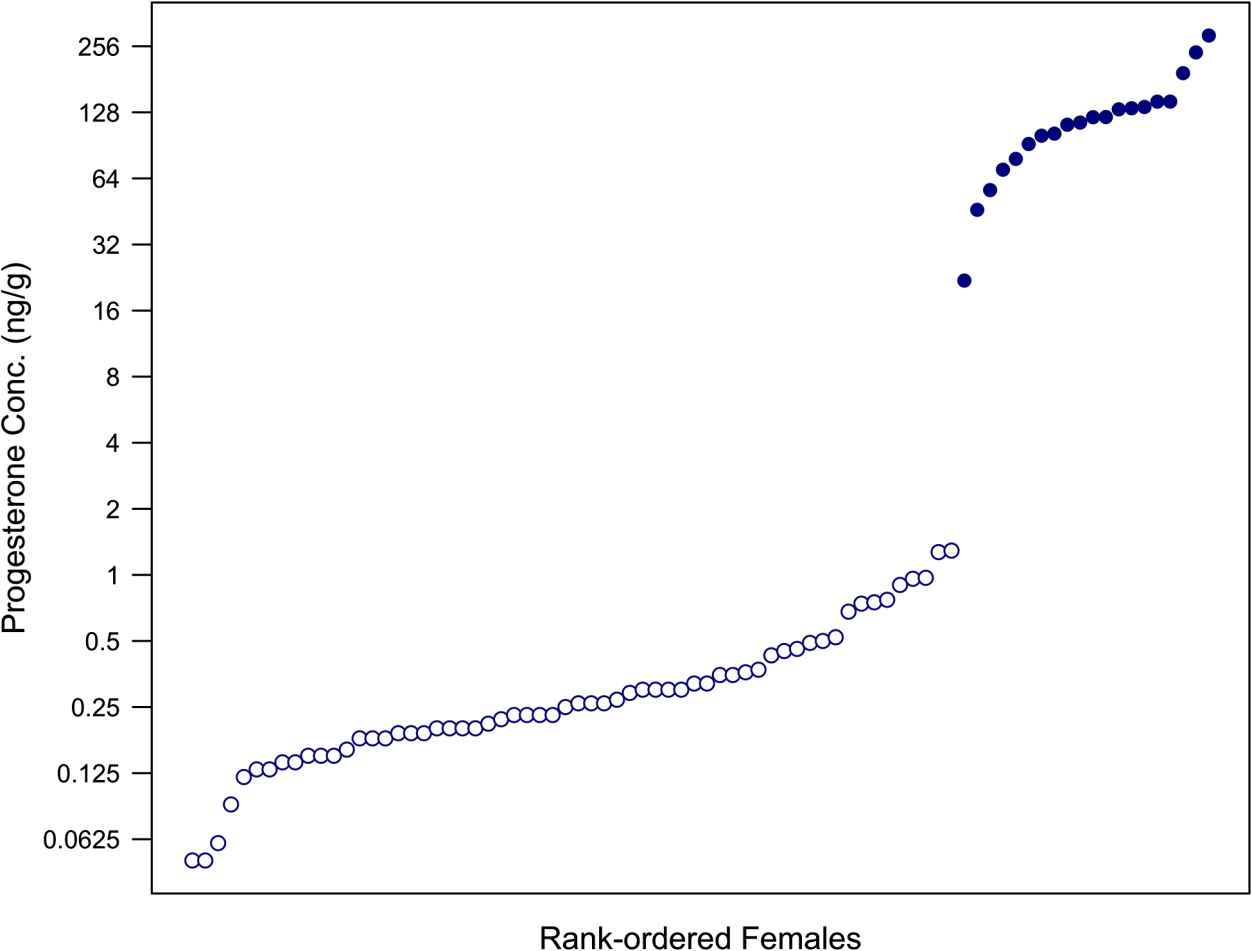
Blubber progesterone concentrations of rank-ordered female humpback whales. The vertical gap towards the right side of the plot illustrates the substantial difference between animals classified as pregnant (filled circles) and those classified as nonpregnant (open circles).

